# Expression of Cathepsins B, D, and G in WHO Grade I Meningioma

**DOI:** 10.3389/fsurg.2019.00006

**Published:** 2019-03-12

**Authors:** Rosanna M. A. Rahman, Bede van Schaijik, Helen D. Brasch, Reginald W. Marsh, Agadha C. Wickremesekera, Reuben Johnson, Kelvin Woon, Swee T. Tan, Tinte Itinteang

**Affiliations:** ^1^Gillies McIndoe Research Institute, Wellington, New Zealand; ^2^Department of Neurosurgery, Wellington Regional Hospital, Wellington, New Zealand; ^3^Faculty of Medicine, Auckland University, Auckland, New Zealand; ^4^Wellington Regional Plastic, Maxillofacial and Burns Unit, Hutt Hospital, Wellington, New Zealand

**Keywords:** meningioma, cathepsin B, cathepsin D, cathepsin G, tumor stem cells, renin-angiotensin system

## Abstract

**Aim:** We have recently demonstrated the presence of putative tumor stem cells (TSCs) in World Health Organization (WHO) grade I meningioma (MG) localized to the microvessels, which expresses components of the renin-angiotensin system (RAS). The RAS is known to be dysregulated and promotes tumorigenesis in many cancer types, including glioblastoma. Cathepsins B, D, and G are isoenzymes that catalyze the production of angiotensin peptides, hence providing bypass loops for the RAS. This study investigated the expression of cathepsins B, D, and G in WHO grade I MG in relation to the putative TSC population we have previously demonstrated.

**Methods:** 3,3-Diaminobenzidine (DAB) immunohistochemical (IHC) staining with antibodies for cathepsins B, D, and G was performed on WHO grade I MG tissue samples from 10 patients. Three of the MG samples subjected to DAB IHC staining underwent immunofluorescence (IF) IHC staining to investigate co-expression of each of these cathepsins using combinations of smooth muscle actin (SMA) and embryonic stem cell marker OCT4. NanoString mRNA expression (*n* = 6) and Western blotting (WB; *n* = 5) analyses, and enzyme activity assays (EAAs; *n* = 3), were performed on snap-frozen WHO grade I MG tissue samples to confirm transcriptional activation, protein expression, and functional activity of these proteins, respectively.

**Results:** DAB IHC staining demonstrated expression of cathepsins B, D, and G in all 10 MG samples. NanoString mRNA expression and WB analyses showed transcriptional activation and protein expression of all three cathepsins, although cathepsin G was expressed at low levels. EAAs demonstrated that cathepsin B and cathepsin D were functionally active. IF IHC staining illustrated localization of cathepsin B and cathepsin D to the endothelium and SMA^+^ pericyte layer of the microvessels, while cathepsin G was localized to cells scattered within the interstitium, away from the microvessels.

**Conclusion:** Cathepsin B and cathepsin D, and to a lesser extent cathepsin G, are expressed in WHO grade I MG. Cathepsin B and cathepsin D are enzymatically active and are localized to the putative TSC population on the microvessels, whereas cathepsin G was localized to cells scattered within the interstitium, These results suggest the presence of bypass loops for the RAS, within WHO grade I MG.

## Introduction

Meningioma (MG) is one of the most common primary neoplasms of the central nervous system with an age-adjusted incidence of 11.5 per 100,000 person-years (1–3). Risk factors for the development of MG include hereditary syndromes such as neurofibromatosis type 2, high-dose radiation, and being female (1, 4–7). The standard of care for MG is gross total resection. However, due to tumor location and/or surgical morbidity, complete resection is not achievable in 50% of cases (8). Incomplete resection along with high recurrence rates of up to 32% during long-term follow-up (8–10) underscore a need for more optimal medical management of MG.

The prevailing concept proposes a neuroectodermal origin of MG, specifically arachnoid cap cells (11). However, there is accumulating evidence for the presence of tumor stem cells (TSCs) within MG (12). Tumorigenesis is proposed to be driven by a subpopulation of TSCs, which originate from acquired mutations in normal resident embryonic stem cells (ESC), progenitor cells or differentiated cells (13–15). Takahashi et al. (16) were the first to elucidate expression of ESC markers OCT4, SALL4, NANOG, SOX2, c-MYC, and KLF4, with the ability to induce and maintain pluripotency in addition to a deregulated capacity for renewal in induced pluripotent stem cells. We have recently demonstrated the presence of an ESC-like population which expresses OCT4, NANOG, SOX2, KLF4, and c-MYC on both the endothelial and pericyte layers of the microvessels in World Health Organization (WHO) grade I MG (17). This ESC-like population expresses components of the renin-angiotensin system (RAS): pro(renin) receptor (PRR), angiotensin converting enzyme (ACE), angiotensin II receptor 1 (ATIIR1), and angiotensin II receptor 2 (ATIIR2) in the microvessels in this tumor (18).

While the RAS is classically associated with regulation of blood pressure and blood volume, it also plays a role in cancer by inducing angiogenesis, promoting cellular proliferation, and inhibiting apoptosis (19–21). The RAS is dysregulated in malignancy, promoting tumor growth in cancers such as non-small cell lung (22), ovarian (23), pancreatic (24), and prostate (25) cancer, as well as brain tumors such as glioblastoma (GB) (26).

**Figure 1:**
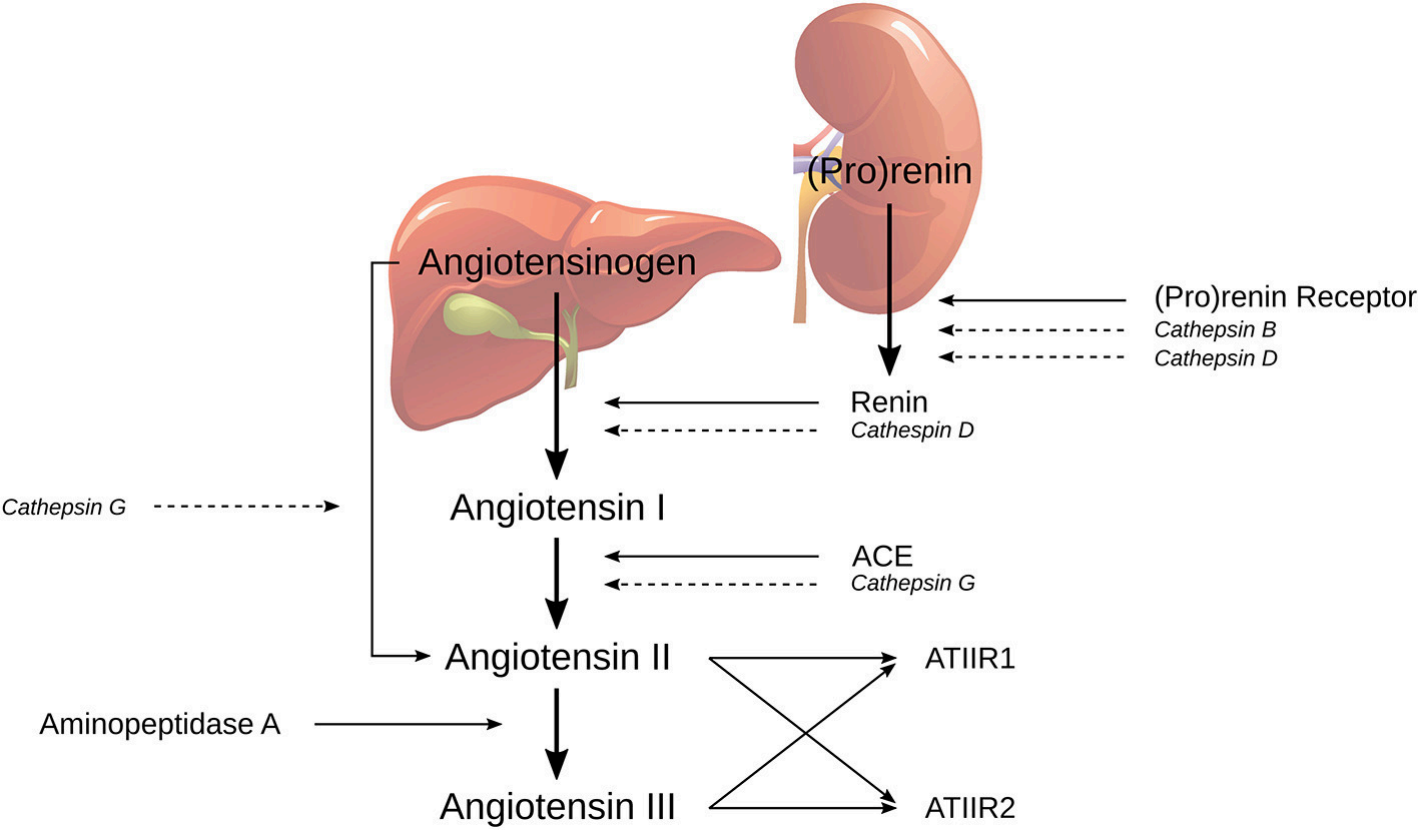
The classical renin-angiotensin system (RAS) and its bypass loops consisting of cathepsins B, D, and G. Classically (pro)renin, from the juxtaglomerular cells in the kidney, is activated by binding to the (pro)renin receptor. Renin coverts angiotensinogen (AGN), produced by the liver, to form angiotensin I (ATI), which is further cleaved to form angiotensin II (ATII), by angiotensin converting enzyme (ACE). Aminopeptidase A then converts ATII to angiotensin III; both of which activate either angiotensin II receptor 1 (ATIIR1) and/or angiotensin II receptor 2 (ATIIR2). Alternately, the RAS may be activated via its bypass loops. Cathepsin B and cathepsin D are renin-activating enzymes. Cathepsin D bears significant homology to renin and is therefore able to independently catalyze the conversion of AGN to form ATI. Cathepsin G may form ATII from either ATI or directly from AGN. Adapted with permission from *Integrative Cancer Science and Therapeutics* (19).

The classical RAS cascade (Figure 1) is initiated by (pro) renin, physiologically secreted from the juxtaglomerular cells in the kidney, and binds to PRR. Renin then catalyzes the conversion of the precursor angiotensinogen (AGN), which is physiologically produced by the liver, to angiotensin I (ATI) (21, 27). ATI is further cleaved by endothelial-bound ACE to form angiotensin II (ATII), which is in turn converted in to angiotensin III (ATIII) by aminopeptidase A. ATII and ATIII can produce vasoconstriction or vasodilatory effects depending on whether they bind to ATIIR1 or ATIIR2, respectively (19).

The RAS can be activated through the classical cascade mechanisms initiated by (pro)renin and PRR or via bypass loops by a group of lysosomal proteases called cathepsins (19). Cathepsin B is a cysteine lysosomal protease that catalyzes the conversion of pro (renin) into active renin without activating the PRR (28). Cathepsin D is an aspartic lysosomal protease that can directly convert AGN to ATI due to its significant homology to renin (29). Cathepsin G is a serine lysosomal protease with the capacity to generate ATII from ATI, or directly from AGN, without the action of renin or ACE (30). Therefore, any attempt at modulating RAS-induced tumorigenesis with traditional RAS modulators such as β-blockers, ACE inhibitors (ACEIs) or angiotensin receptor blockers (ARBs), may be circumvented by cathepsin-induced activation of the RAS.

The expression of cathepsin B by the stem cell *niche* on the microvessels in GB has been reported recently (31), with this staining pattern being a negative prognostic factor (32). This study was aimed at investigating the expression of cathepsins B, D, and G in WHO grade I MG, in relation to the putative TSC population we have previously identified (17).

## Materials and Methods

### Tissue Samples

WHO grade I MG tissue samples from one male and nine female patients, age 36–85 (mean, 61.2) years included in our previous studies (17, 18), were sourced from the Gillies McIndoe Research Institute Tissue Bank for this study. Study approval was granted by the Central Regional Health and Disability Ethics Committee (Ref. no 15CEN28). Informed written consent was obtained from all patients.

### Histochemical and Immunohistochemical Staining

The diagnosis of WHO grade I MG was confirmed by an anatomical pathologist (HDB) using hematoxylin and eosin (H&E) stained 4 μm-thick formalin-fixed paraffin-embedded sections of MG samples from all 10 patients. 3,3-Diaminobenzidine (DAB) and immunofluorescent (IF) immunohistochemical (IHC) staining was performed, as previously described (33, 34), on the same MG samples. The staining was completed on the Leica Bond Rx auto-stainer (Leica, Nussloch, Germany) with the primary antibodies: cathepsin B (1:1,000; cat# sc-6490-R, Santa Cruz, CA, USA), cathepsin D (1:200; cat# NCL-CDm, Leica), cathepsin G (1:200; cat# sc-33206, Santa Cruz), smooth muscle actin (SMA; ready to use; cat# PA0943, Leica), and OCT4 (1:30; cat# MRQ-10, Cell Marque, Rocklin, CA, USA). All antibodies were diluted with Bond™ primary antibody diluent (cat# AR9352, Leica).

To further characterize the expression of cathepsins B, D, and G, IF IHC staining was performed on three representative MG samples from the original cohort of 10 patients included in the DAB IHC staining. Dual IF IHC staining was performed for each of the cathepsins using identical primary antibodies and concentrations as for DAB IHC staining, in conjunction with SMA or OCT4. An appropriate fluorescent secondary antibody combination of Vectafluor Excel anti-rabbit 594 (ready-to-use, cat# VEDK-1594, Vector Laboratories, Burlingame, CA, USA) and Alexa Fluor anti-mouse 488 (1:500, cat# A21202, Life Technologies) was used for detection.

DAB IHC-stained slides were counterstained with hematoxylin and mounted in Surgipath Micromount mounting medium (cat# 3801732, Leica), whereas IF IHC-stained slides were mounted in Vectashield HardSet anti-fade mounting medium and counter-stained with 4′6-diamino-2-phenylinodole (cat# H-1500, Vector Laboratories).

Positive controls for DAB IHC staining included human placenta for cathepsin B, human breast tissue for cathepsin D and human splenic lymphoma for cathepsin G. Negative controls were performed on sections of WHO grade I MG using a matched isotype control for rabbit primary antibodies (ready-to-use; cat# IR600, Dako), to determine the specificity of the antibody. Negative controls for IF IHC staining were performed using a section of MG tissue with the combined use of primary isotype mouse (ready-to-use; cat# IR750, Dako) and rabbit (ready-to-use; cat# IR600, Dako) antibodies.

### Image Analysis

The DAB IHC-stained slides were viewed and images were captured, using an Olympus BX53 light microscope fitted with an Olympus DP21 digital camera and processed with CellSens 2.0 software (Olympus, Tokyo, Japan). The IF IHC-stained images were captured using the Olympus FV1200 biological confocal laser-scanning microscope and processed with CellSens Dimension 1.11 software using the 2D deconvolution algorithm (Olympus).

### Western Blotting

Western blot (WB) analysis was performed on five snap-frozen WHO grade I MG samples from the original cohort of 10 patients included for DAB IHC staining. Total protein was extracted and precipitated from the tissue samples, as previously reported (35), followed by gel electrophoresis on a 4–12% 1D PAGE (cat# NW04120BOX, Thermo Fisher Scientific) with transfer to a PVDF membrane (cat# IB24001, Invitrogen, Carlsbad, CA, USA) using an iBlot 2 (cat# IB21001, Thermo Fisher Scientific). Detection of the proteins was performed on the iBind Flex (cat# SLF2000, Thermo Fisher Scientific) using the primary antibodies for cathepsin B (1:250; cat# SC-6490-R, Santa Cruz), cathepsin D (1:250; cat# SC-6486, Santa Cruz), cathepsin G (1:250; cat# ab197354, Abcam, Cambridge, UK), and α-tubulin (1:1000; cat# 62204, Invitrogen). Appropriate secondary antibodies were goat anti-rabbit Alexa Fluor 647 (1:2000; cat# A21244, Life Technologies) for cathepsins B, D, and G and goat anti-mouse Alexa Fluor 488 (1:2000; cat# A21202, Life Technologies) for α-tubulin. WB bands were detected by the Chemi Doc MP Imaging System (Bio-Rad) and converted in to densitometry readings and analyzed using Image Lab 6.0 software (Bio-Rad). All experiments were performed in triplicate. Snap-frozen human tonsillar tissue was used as positive control tissue for cathepsin B and cathepsin D, with a recombinant cathepsin G protein (cat# H00001511-Q01, Novus Biologicals, Littleton, CO, USA) as the positive control for cathepsin G. Matched mouse (1:500; cat# ab18443, Abcam) and rabbit (1:500; cat# ab171870, Abcam) isotype primary antibodies were used as appropriate negative controls.

### Enzymatic Activity Assays

Enzymatic activity of cathepsin B and cathepsin D were determined in snap-frozen WHO grade I MG tissue samples from three of the five samples used for WB analysis. Enzymatic activity assay (EAA) kits were utilized for cathepsin B (cat# ab65300; Abcam) and cathepsin D (cat# ab65302; Abcam). All steps of the procedure were performed according to the manufacturer's protocol, and as recently described (33). Fluorescence was measured in a Nunc^TM^ F96 MicroWell^TM^ black polystyrene plate (cat# 136101, Thermo Fisher Scientific) using the Varioskan Flash plate reader (cat# MIB5250030, Thermo Fisher Scientific). Tonsil and denatured tonsil tissue lysates were used as appropriate positive and negative controls, respectively.

### NanoString mRNA Expression Analysis

mRNA expression of cathepsins B, D, and G was determined by NanoString mRNA analysis in six snap-frozen WHO grade I MG samples of the original cohort of 10 patients used for DAB IHC staining. Total RNA was extracted separately from ~20 mg of each snap-frozen MG tissue and run through the NanoString nCounter™ Gene Expression Assay (NanoString Technologies, Seattle, WA, USA), as previously described (27, 36). Probes for the genes encoding cathepsin B (NM_001908.2), cathepsin D (NM_001909.3) cathepsin G (NM_001911.2) and the housekeeping gene GUSB (NM_000181.1), were designed and synthesized by NanoString Technologies. Raw data were analyzed with nSolver™ software (NanoString Technologies) using standard settings and normalized against the housekeeping gene.

### Statistical Analyses

Data obtained from WB and NanoString mRNA analyses were subjected to paired *t*-tests using SPSS version 24 software.

## Results

### Histochemical and DAB IHC Staining

**Figure 2:**
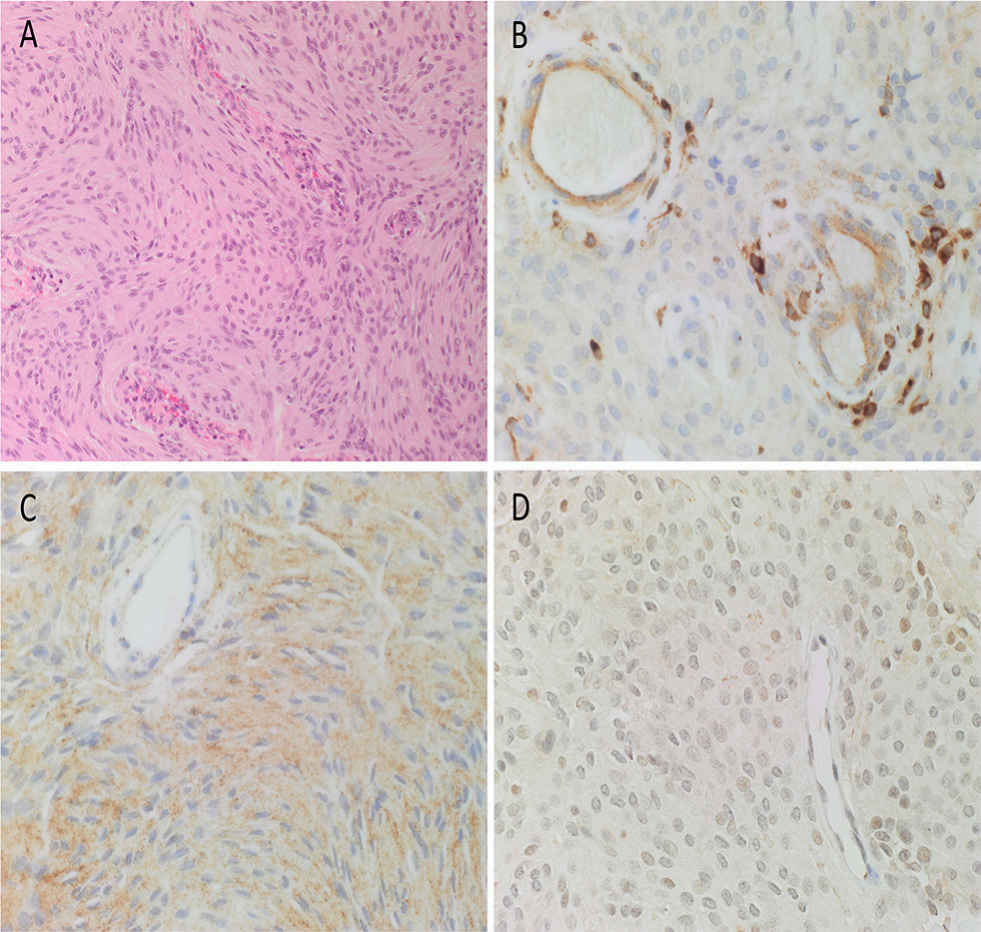
Representative hematoxylin and eosin-stained section of a WHO grade I meningioma (MG) demonstrating the distinctive and characteristic whorls **(A)**. Representative 3,3-diaminobenzidine immunohistochemical-stained sections of WHO grade I MG demonstrating cytoplasmic and/or nuclear expression of cathepsins B (**B**, brown), D (**C**, brown), and G (**D**, brown). Cell nuclei were stained with hematoxylin (**A–D**, blue). Original magnification: **(A)** 200x; **(B)** 400x.

The diagnosis of WHO grade I MG was confirmed by H&E staining for all 10 tissue samples (Figure 2A). All DAB IHC-stained sections exhibited ubiquitous, granular, cytoplasmic staining for cathepsin B (Figure 2B, brown) and cathepsin D (Figure 2C, brown). Cytoplasmic and nuclear staining for cathepsin G (Figure 2D, brown) showed scattered clusters of focally positive cells.

The expected positive staining patterns for cathepsin B (Supplementary Figure 1A, brown), cathepsin D (Supplementary Figure 1B, brown), and cathepsin G (Supplementary Figure 1C, brown) were demonstrated on human placenta tissue, human breast tissue, and human splenic lymphoma, respectively. Negative control tissues showed minimal staining for all three cathepsins on sections of WHO grade I MG tissue using a matched isotype control for both mouse and rabbit primary antibodies (Supplementary Figure 1D, brown).

### IF IHC Staining

**Figure 3:**
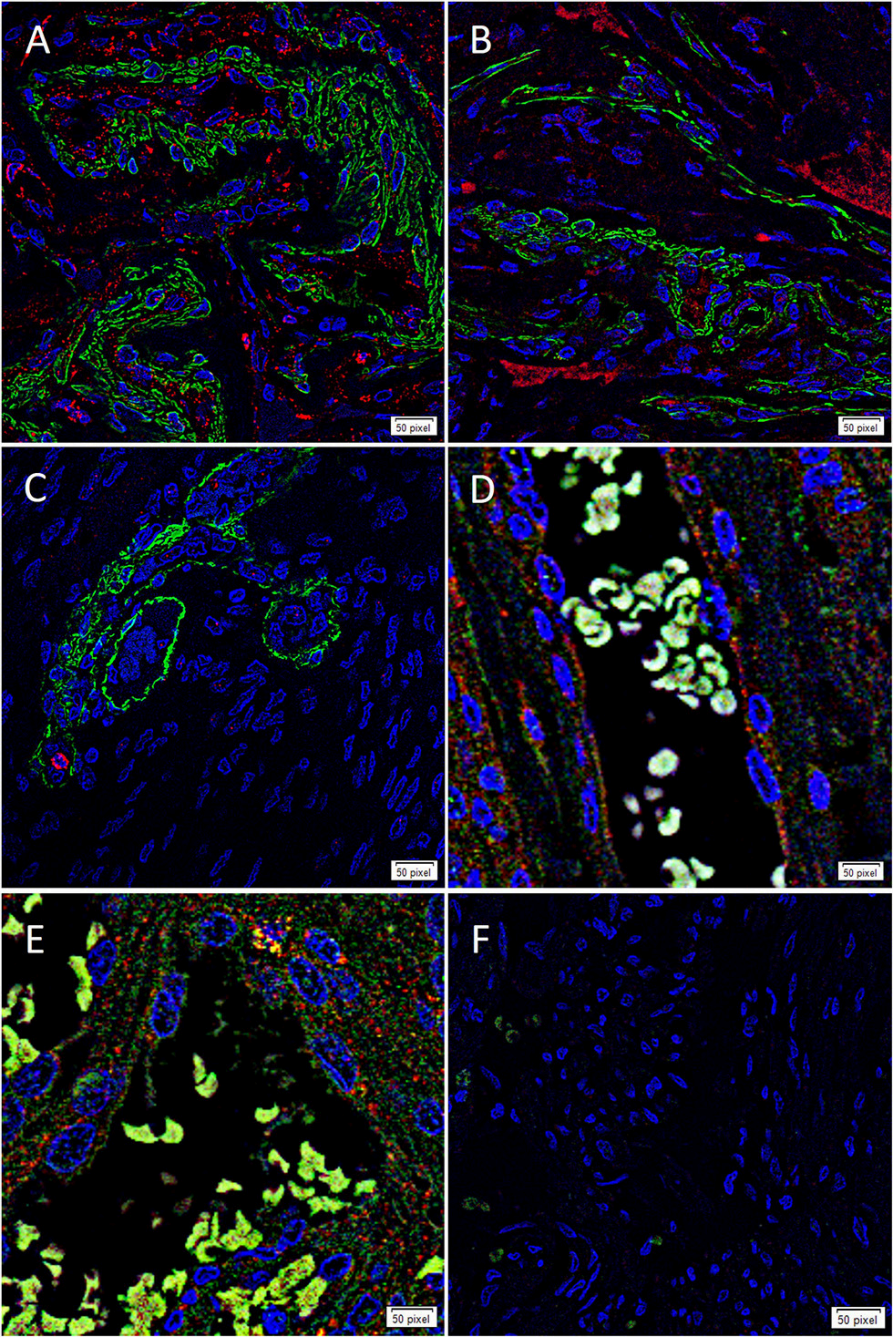
Representative immunofluorescence immunohistochemical staining of WHO grade I meningioma demonstrating the expression of cathepsin B (**A,D**, red) and cathepsin D (**B,E**, red) on the endothelium and the smooth muscle actin^+^ (SMA; **A,B**, green) outer pericyte layer. Cathepsin G (**C**, red) was expressed by cells outside the microvessels with the outer layer stained positively for SMA. The expression of both cathepsin B and cathepsin D was also demonstrated with the embryonic stem cell marker OCT4 (**D,E**, green). A negative control **(F)** to test the specificity of the fluorescent secondary antibodies was performed on a section of MG. Cell nuclei were counterstained with 4′,6-diamidino-2-phenylindole (**A–F**, blue). Scale bar: 50μm.

To determine whether each cathepsin was expressed by the putative TSC population on the microvessels that express the ESC markers we have previously reported in WHO grade I MG (17), we co-stained each cathepsin with the pericyte marker, SMA (37). Cathepsin B (Figure 3A, red) and cathepsin D (Figure 3B, red) were illustrated on both the endothelium as well as the outer pericyte layer. These were in contrast to cathepsin G (Figure 3C, red), which was expressed by stained cells away from the microvessels.

To confirm the expression of both cathepsin B (Figure 3D, red) and cathepsin D (Figure 3E, red) on the putative TSC population, we performed co-staining with the stem cell marker, OCT4 (Figures 3D,E, green), confirming co-expression of both markers in the microvessels. Negative isotype controls demonstrated minimal staining as expected (Figure 2F).

### Western Blotting

**Figure 4:**
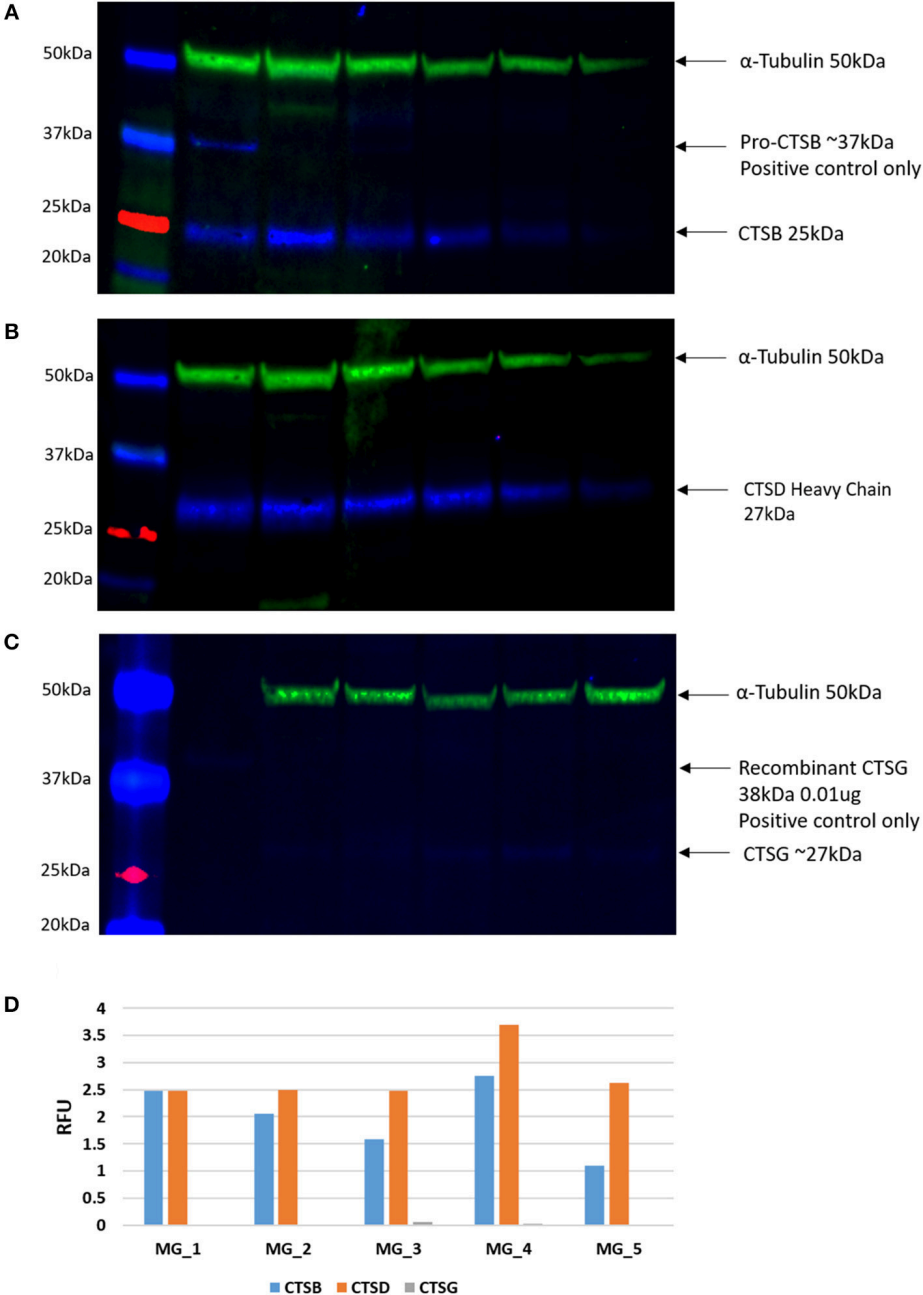
Individual Western blotting results for cathepsin B (CTSB) **(A)**, cathespsin D (CTSD) **(B)** and **(C)** in six WHO grade I meningioma (MG) samples. The approximate protein loading equivalent for all MG samples was confirmed with α-tubulin **(A–C)**. Densitometry results demonstrated the average intensity of each blot, by cathepsin, expressed in relative fluorescence units (RFU; **D**).

WB of the snap-frozen WHO grade I MG samples demonstrated the presence of bands at the expected molecular weight of 25 kDa for cathepsin B (Figure 4A, blue) and 28 kDa for cathepsin D (Figure 4B, blue) in all five MG samples. Cathepsin G was also detected at the expected molecular weight of 29 kDa (Figure 4C, blue), but at relatively low levels in all five samples. α-tubulin confirmed approximate equivalent protein loading for the MG samples examined (Figures 4A–C, green). Densitometry was performed to convert the WB data in to numerical values for quantitative analysis. Results for cathepsins B, D, and G (Figure 4D), relative to α-tubulin, confirmed significant differences in the relative abundance of cathepsin B (*p* < 0.002) and cathepsin D (*p* <0.0001) compared to cathepsin G.

### Enzymatic Activity Assays

**Figure 5:**
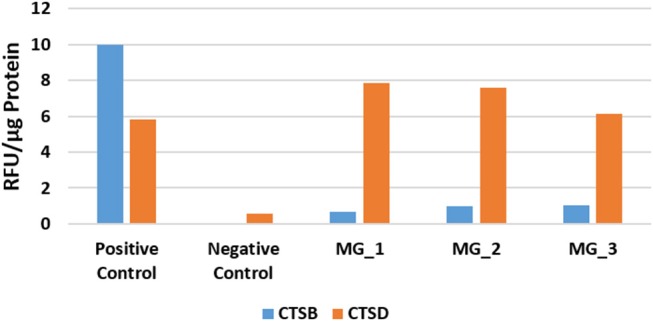
Averaged results of cathepsin B (CTSB) and cathepsin D (CTSD) for three WHO grade I meningioma samples expressed in relative fluorescence units (RFU)/ug of protein, compared to the positive and negative controls.

In light of the WB analysis, detecting only cathepsin B and cathepsin D, we limited our study to determine the functional activity of these two cathepsins. EAAs demonstrated enzymatic activity for cathepsin B and cathepsin D (Figure 5).

### NanoString mRNA Expression Analysis

**Figure 6:**
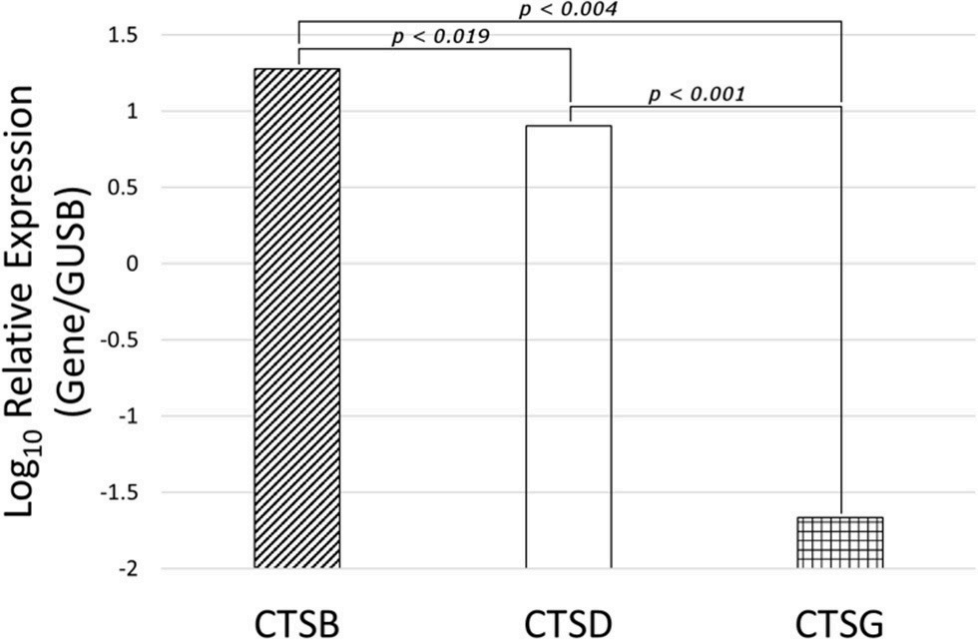
Expression of cathepsins cathespsin B (CTSB), cathepsin D (CTSD) and cathepsin G (CTSG) mRNA transcripts in WHO grade I meningioma samples from six patients, normalized over the housekeeping gene GUSB and presented as Log_10_ Relative Expression. Paired *t*-tests demonstrated significantly higher levels of expression of cathepsin B (*p* < 0.004) and cathepsin D (*p* < 0.001) than cathepsin G. Cathepsin B and cathepsin D were only marginally different to each other (*p* > 0.019).

NanoString mRNA expression analysis demonstrated the presence of mRNA transcripts for cathepsins B, D, and G, normalized against the housekeeping gene GUSB, in all six MG samples (Figure 6). Statistical analysis of the NanoString data, using paired *t*-tests, further confirmed that the mean levels of expression of cathepsin B (*p* <0.004) and cathepsin D (*p* <0.001) were significantly higher than that of cathepsin G, but only marginally different to each other (*p* <0.019).

## Discussion

This study demonstrated the expression of cathepsins B, D, and G in WHO grade I MG. Cathepsin B and cathepsin D were enzymatically active and were demonstrated on the putative TSC population of the endothelial and pericyte layers of the microvessels (17), which expresses components of the RAS (18). NanoString mRNA expression and WB analyses demonstrated low levels of mRNA transcripts and protein expression of cathepsin G, respectively. DAB and IF IHC staining showed that this protein was expressed by cells scattered within the interstitium, away from the microvessels in the WHO grade I MG samples examined. It is interesting to speculate that the cathepsin G^+^ cells may be of a mast cell phenotype, as previously reported (38); however, this topic requires further investigation.

The faint OCT4 IF IHC staining maybe due to the relative low abundance of this protein, and the use of dual DAB IHC staining could further accentuate the staining. Furthermore, the relative abundant staining for SMA, maybe more representative of arterioles or venules.

Elevated expression of cathepsin B and cathepsin D has been observed in many tumor types such as infantile hemangioma (38), oral tongue squamous cell carcinoma (33), MG (39), and gliomas (40, 41) including GB (34). Interestingly, the cathepsin B expression pattern that we report here almost parallels that reported with GB cancer stem cells (31). Taken together, upregulation of cathepsin B within WHO grade 1 MG may indicate a growth phase in this tumor, although, this requires further investigation.

Research into the role of cathepsin B and cathepsin D in tumorigenesis, so far, has mainly focused on their independent influence on tumor invasiveness. Malignant transformation results in the overproduction of cathepsin B (42) and elevated cathepsin B levels correlate with increased invasiveness and recurrence of MG (32, 39, 43, 44), astrocytoma and gliomas (39, 45). As an intracellular lysosomal protease, cathepsin B has been found in the extracellular matrix (ECM) of colorectal cancer, localized to the invasive front of the tumor (46). The involvement of cathepsin B in tissue invasion has been attributed to its proteolytic activation of ECM components urokinase plasminogen activator, plasminogen and plasmin, which further activate metalloproteinases (MMPs), whilst destroying MMP inhibitors, thereby enabling ECM degradation (47). At the same time cathepsin B promotes autophagy and cannibalism allowing tumor cells to recycle nutrients and maintain a proliferative and infiltrative phenotype (42). Despite increasing evidence for its involvement in promoting invasive tumor grades, little is known about the role of cathepsin B in its RAS-related influence on tumorigenesis in non-invasive WHO grade I MG, which represent 80% of all tumors (48).

Evidence of a role for cathepsin D in brain tumors, particularly MG, has so far been inconclusive (49). Some studies have found elevated levels of cathepsin D in high-grade astrocytomas (45) and GB (34, 50), while others have shown no association with invasive MG (51) or an inverse relationship between cathepsin D levels and MG tumor grade (52). Despite the ability of cathepsin G to activate the RAS via multiple bypass loops, there is a paucity of literature on its potential role in tumorigenesis. This study demonstrated low expression of cathepsin G, which was localized to cells scattered within the interstitium, away from the microvessels, in WHO grade I MG.

The RAS is known to be dysregulated in malignancy and promote tumorigenesis in many cancer types. The expression of cathepsins B, D, and G in WHO grade I MG suggests the presence of bypass loops for the RAS, which could potentially negate the effectiveness of traditional RAS modulators such as β-blockers, ACEIs and ARBs (30, 53, 54). We have previously demonstrated the putative presence of an ESC-like population localized to the microvessels within WHO grade I MG (12, 17), that expresses components of the RAS (18). This study demonstrates that cathepsin B and cathepsin D and possibly cathepsin G, are also expressed and cathepsin B and cathepsin D are active in the same TSCs in WHO grade I MG.

Our results suggest that cathepsin B and cathepsin D may be novel therapeutic targets, in addition to the classical RAS, for WHO grade I MG. Limitations of the study include the relatively small sample size. Future studies including a larger sample size and functional *in vivo* experiments are needed to conclusively determine the role of these proteases within MG. In addition, more distinct cellular staining could be improved using chromogenic DAB IHC staining to localize the markers investigated.

## Author Contributions

TI and ST formulated the study hypothesis. TI, AW, and ST designed the study. AW, KW, and RJ recruited patients and obtained study samples. TI, HB, AW, RR, and ST interpreted the DAB IHC data. TI, RR, and ST interpreted the IF IHC data. BvS performed the WB analysis. BvS, TI, AW, and ST interpreted the WB data. TI, RR, and ST interpreted the NanoString mRNA analysis data. RM carried out statistical analysis. RR, BvS, AW, TI, and ST drafted the manuscript. All authors commented on and approved the manuscript.

### Conflict of Interest Statement

TI and ST are inventors of the PCT patents Cancer Diagnosis and Therapy (PCT/NZ2015/050108) and Cancer Therapeutic (PCT/NZ2018/050006), and provisional patent application Novel Pharmaceutical Compositions for Cancer Therapy (US/62/711709). The remaining authors declare that the research was conducted in the absence of any commercial or financial relationships that could be construed as a potential conflict of interest.

## Supplementary Material

Supplementary Figure 1 Representative 3,3-diaminobenzidine (DAB) immunohistochemical (IHC)-stained sections of positive human control tissues: placenta tissue for cathepsin B **(A)**, breast tissue for cathepsin D **(B)**, and splenic lymphoma for cathepsin G **(C)**. A representative negative control section of WHO grade I meningioma tissue using a matched isotype control for both mouse and rabbit primary antibodies showed minimal staining **(D)**. Original magnification: 400x.

## References

[1] Wiemels J Wrensch M Claus EB. Epidemiology and etiology of meningioma. J Neurooncol. (2010) 99:307–14. 10.1007/s11060-010-0386-3 20821343 PMC2945461

[2] Ostrom QT Barnholtz-Sloan JS. Current state of our knowledge on brain tumor epidemiology. Curr Neurol Neurosci Rep. (2011) 11:329–35. 10.1007/s11910-011-0189-8 21336822

[3] Goldbrunner R Minniti G Preusser M Jenkinson MD Sallabanda K Houdart E. EANO guidelines for the diagnosis and treatment of meningiomas. Lancet Oncol. (2016) 17:e383–91. 10.1016/S1470-2045(16)30321-7 27599143

[4] Bondy ML Scheurer ME Malmer B Barnholtz-Sloan JS Davis FG Il'yasova D. Brain tumor epidemiology: consensus from the Brain Tumor Epidemiology Consortium. Cancer. (2008) 113(Suppl. 7):1953–68. 10.1002/cncr.23741 18798534 PMC2861559

[5] Mealey J Carter JE. Spinal cord tumor during pregnancy. Obstet Gynecol. (1968) 32:204–9. 5742100

[6] Rausing A Ybo W Stenflo J. Intracranial meningioma–a population study of ten years. Acta Neurol Scand. (1970) 46:102–10. 10.1111/j.1600-0404.1970.tb05608.x 5412623

[7] Earle KM Richany SF. Meningiomas. A study of the histology, incidence, and biologic behavior of 243 cases from the Frazier-Grant collection of brain tumors. Med Ann Dist Columbia. (1969) 38:353–6 passim. 5255150

[8] De La Garza-Ramos R Flores-Rodríguez JV Martínez-Gutiérrez JC Ruiz-Valls A Caro-Osorio E. Current standing and frontiers of gene therapy for meningiomas. Neurosurg Focus. (2013) 35:E4. 10.3171/2013.8.FOCUS13305 24289129

[9] Fathi AR Roelcke U. Meningioma. Curr Neurol Neurosci Rep. (2013) 13:337. 10.1007/s11910-013-0337-4 23463172

[10] Kim MS Yu DW Jung YJ Kim SW Chang CH Kim OL. Long-term follow-up result of hydroxyurea chemotherapy for recurrent meningiomas. J Korean Neurosurg Soc. (2012) 52:517–22. 10.3340/jkns.2012.52.6.517 23346322 PMC3550418

[11] Albayrak SB Black PML The origin of meningiomas. In: Pamir MN Black PML Fahlbusch R editors. Meningiomas: A Comprehensive Text. Saunders/Elsevier (2010). p. 53.

[12] Shivapathasundram G Wickremesekera AC Tan ST Itinteang T. Tumour stem cells in meningioma: a review. J Clin Neurosci. (2018) 47:66–71. 10.1016/j.jocn.2017.10.059 29113852

[13] Goldthwaite CA Regenerative Medicine. Bethesda, MD: National Institutes of Health, U.S. Department of Health and Human Services (2006).

[14] Kreso A Dick JE. Evolution of the cancer stem cell model. Cell Stem Cell. (2014) 14:275–91. 10.1016/j.stem.2014.02.006 24607403

[15] Singh SK Clarke ID Terasaki M Bonn VE Hawkins C Squire J. Identification of a cancer stem cell in human brain tumors. Cancer Res. (2003) 63:5821–8. 14522905

[16] Takahashi K Tanabe K Ohnuki M Narita M Ichisaka T Tomoda K. Induction of pluripotent stem cells from adult human fibroblasts by defined factors. Cell. (2007) 131:861–72. 10.1016/j.cell.2007.11.019 18035408

[17] Shivapathasundram G Wickremesekera A Brasch H Marsh R Tan S Itinteang T Expression of embryonic stem cell markers on the microvessels of grade 1 meningioma. Front Surg. (2018) 5:65 10.3389/fsurg.2018.00065 30417000 PMC6212465

[18] Shivapathasundram G Wickremesekera A Brasch H van Schaijik B Marsh R Tan S Expression of components of the renin-angiotensin system by the putative stem cell population in WHO grade I meningioma. Front Surg. (2019) 5:65 10.3389/fsurg.2019.00006 PMC6532688 31157231

[19] Munro MJ Wickremesekera AC Davis PF Marsh R Tan ST Itinteang T Renin-angiotensin system and cancer: a review. Integr Cancer Sci Ther. (2017) 4:1–6. 10.15761/ICST.1000231

[20] Ager EI Neo J Christophi C. The renin-angiotensin system and malignancy. Carcinogenesis. (2008) 29:1675–84. 10.1093/carcin/bgn171 18632755

[21] George AJ Thomas WG Hannan RD. The renin-angiotensin system and cancer: old dog, new tricks. Nat Rev Cancer. (2010) 10:745–59. 10.1038/nrc2945 20966920

[22] Feng Y Wan H Liu J Zhang R Ma Q Han B. The angiotensin-converting enzyme 2 in tumor growth and tumor-associated angiogenesis in non-small cell lung cancer. Oncol Rep. (2010) 23:941–8. 10.3892/or_00000718 20204277

[23] Ino K Shibata K Kajiyama H Yamamoto E Nagasaka T Nawa A. Angiotensin II type 1 receptor expression in ovarian cancer and its correlation with tumour angiogenesis and patient survival. Br J Cancer. (2006) 94:552–60. 10.1038/sj.bjc.6602961 16434990 PMC2361172

[24] Doi C Egashira N Kawabata A Maurya DK Ohta N Uppalapati D. Angiotensin II type 2 receptor signaling significantly attenuates growth of murine pancreatic carcinoma grafts in syngeneic mice. BMC Cancer. (2010) 10:67. 10.1186/1471-2407-10-67 20181281 PMC2846883

[25] Li H Qi Y Li C Braseth LN Gao Y Shabashvili AE. Angiotensin type 2 receptor-mediated apoptosis of human prostate cancer cells. Mol Cancer Ther. (2009) 8:3255–65. 10.1158/1535-7163.MCT-09-0237 19996275

[26] Juillerat-Jeanneret L Celerier J Chapuis Bernasconi C Nguyen G Wostl W Maerki HP. Renin and angiotensinogen expression and functions in growth and apoptosis of human glioblastoma. Br J Cancer. (2004) 90:1059–68. 10.1038/sj.bjc.6601646 14997208 PMC2409624

[27] Bradshaw AR Wickremesekera AC Brasch HD Chibnall AM Davis PF Tan ST. Glioblastoma multiforme cancer stem cells express components of the renin-angiotensin system. Front Surg. (2016) 3:51. 10.3389/fsurg.2016.00051 27730123 PMC5037176

[28] Neves FA Duncan KG Baxter JD. Cathepsin B is a prorenin processing enzyme. Hypertension. (1996) 27(3 Pt 2):514–7. 10.1161/hyp.27.3.514 8613195

[29] Hackenthal E Hackenthal R Hilgenfeldt U. Isorenin, pseudorenin, cathepsin D and renin. A comparative enzymatic study of angiotensin-forming enzymes. Biochim Biophys Acta. (1978) 522:574–88. 10.1016/0005-2744(78)90089-X 623774

[30] Rykl J Thiemann J Kurzawski S Pohl T Gobom J Zidek W. Renal cathepsin G and angiotensin II generation. J Hypertens. (2006) 24:1797–807. 10.1097/01.hjh.0000242404.91332.be 16915029

[31] Breznik B Limbaeck Stokin C Kos J Khurshed M Hira VVV Bošnjak R. Cysteine cathepsins B, X and K expression in peri-arteriolar glioblastoma stem cell niches. J Mol Histol. (2018) 49:481–97. 10.1007/s10735-018-9787-y 30046941 PMC6182580

[32] Breznik B Limback C Porcnik A Blejec A Krajnc MK Bosnjak R. Localization patterns of cathepsins K and X and their predictive value in glioblastoma. Radiol Oncol. (2018) 52:433–42. 10.2478/raon-2018-0040 30367810 PMC6287179

[33] Featherston T Marsh RW van Schaijik B Brasch HD Tan ST Itinteang T. Expression and localization of cathepsins B, D, and G in two cancer stem cell subpopulations in moderately differentiated oral tongue squamous cell carcinoma. Front Med. (2017) 4:100. 10.3389/fmed.2017.00100 28775982 PMC5517773

[34] Koh SP Wickremesekera AC Brasch HD Marsh R Tan ST Itinteang T. Expression of cathepsins B, D, and G in isocitrate dehydrogenase-wildtype glioblastoma. Front Surg. (2017) 4:28. 10.3389/fsurg.2017.00028 28611989 PMC5447023

[35] Tan K Brasch HD van Schaijik B Armstrong JR Marsh RW Davis PF. Expression and localization of cathepsins B, D, and G in dupuytren's disease. Plast Reconstr Surg Glob Open. (2018) 6:e1686. 10.1097/GOX.0000000000001686 29616179 PMC5865920

[36] Bradshaw A Wickremesekera A Brasch HD Chibnall AM Davis PF Tan ST Cancer stem cells in glioblastoma multiforme. Front Surg. (2016) 3:48 10.3389/fsurg.2016.00021 27617262 PMC5001191

[37] Itinteang T Tan ST Brasch H Day DJ. Haemogenic endothelium in infantile haemangioma. J Clin Pathol. (2010) 63:982–6. 10.1136/jcp.2010.081257 20924092

[38] Itinteang T Chudakova DA Dunne JC Davis PF Tan ST. Expression of Cathepsins B, D, and G in infantile hemangioma. Front Surg. (2015) 2:26. 10.3389/fsurg.2015.00026 26137466 PMC4470331

[39] Vranic A. Antigen expression on recurrent meningioma cells. Radiol Oncol. (2010) 44:107–12. 10.2478/v10019-010-0028-6 22933900 PMC3423683

[40] Mikkelsen T Yan PS Ho KL Sameni M Sloane BF Rosenblum ML. Immunolocalization of cathepsin B in human glioma: implications for tumor invasion and angiogenesis. J Neurosurg. (1995) 83:285–90. 7542317 10.3171/jns.1995.83.2.0285

[41] Fukuda ME Iwadate Y Machida T Hiwasa T Nimura Y Nagai Y. Cathepsin D is a potential serum marker for poor prognosis in glioma patients. Cancer Res. (2005) 65:5190–4. 10.1158/0008-5472.CAN-04-4134 15958563

[42] Gondi CS Rao JS. Cathepsin B as a cancer target. Expert Opin Ther Targets. (2013) 17:281–91. 10.1517/14728222.2013.740461 23293836 PMC3587140

[43] Tummalapalli P Spomar D Gondi CS Olivero WC Gujrati M Dinh DH. RNAi-mediated abrogation of cathepsin B and MMP-9 gene expression in a malignant meningioma cell line leads to decreased tumor growth, invasion and angiogenesis. Int J Oncol. (2007) 31:1039–50. 10.3892/ijo.31.5.1039 17912429 PMC2031211

[44] Lah TT Nanni I Trinkaus M Metellus P Dussert C De Ridder L. Toward understanding recurrent meningioma: the potential role of lysosomal cysteine proteases and their inhibitors. J Neurosurg. (2010) 112:940–50. 10.3171/2009.7.JNS081729 19747051

[45] Levicar N Strojnik T Kos J Dewey RA Pilkington GJ Lah TT. Lysosomal enzymes, cathepsins in brain tumour invasion. J Neurooncol. (2002) 58:21–32. 10.1023/A:1015892911420 12160137

[46] Guzinska-Ustymowicz K. MMP-9 and cathepsin B expression in tumor budding as an indicator of a more aggressive phenotype of colorectal cancer (CRC). Anticancer Res. (2006) 26:1589–94. 16619576

[47] Kostoulas G Lang A Nagase H Baici A. Stimulation of angiogenesis through cathepsin B inactivation of the tissue inhibitors of matrix metalloproteinases. FEBS Lett. (1999) 455:286–90. 10.1016/S0014-5793(99)00897-2 10437790

[48] Riemenschneider MJ Perry A Reifenberger G. Histological classification and molecular genetics of meningiomas. Lancet Neurol. (2006) 5:1045–54. 10.1016/S1474-4422(06)70625-1 17110285

[49] Castilla EA Prayson RA Abramovich CM Cohen ML. Immunohistochemical expression of cathepsin D in meningiomas. Am J Clin Pathol. (2003) 119:123–8. 10.1309/W0H705HAJL73T0EQ 12520707

[50] Sivaparvathi M Sawaya R Chintala SK Go Y Gokaslan ZL Rao JS. Expression of cathepsin D during the progression of human gliomas. Neurosci Lett. (1996) 208:171–4. 10.1016/0304-3940(96)12584-2 8733297

[51] Backer-Grøndahl T Moen BH Arnli MB Torseth K Torp SH. Immunohistochemical characterization of brain-invasive meningiomas. Int J Clin Exp Pathol. (2014) 7:7206–19. 25400818 PMC4230100

[52] Lusis EA Chicoine MR Perry A. High throughput screening of meningioma biomarkers using a tissue microarray. J Neurooncol. (2005) 73:219–23. 10.1007/s11060-004-5233-y 15980972

[53] Tan ST Itinteang T Leadbitter P. Low-dose propranolol for infantile haemangioma. J Plast Reconstr Aesthet Surg. (2011) 64:292–9. 10.1016/j.bjps.2010.06.010 20615772

[54] Itinteang T Brasch HD Tan ST Day DJ. Expression of components of the renin-angiotensin system in proliferating infantile haemangioma may account for the propranolol-induced accelerated involution. J Plast Reconstr Aesthet Surg. (2011) 64:759–65. 10.1016/j.bjps.2010.08.039 20870476

